# Patricia Lacks' contributions to evidence-based practice for insomnia

**DOI:** 10.3389/frsle.2023.1125054

**Published:** 2023-02-23

**Authors:** Daniel B. Kay, Amy R. Wolfson

**Affiliations:** ^1^Department of Psychology, Brigham Young University, Provo, UT, United States; ^2^Department of Psychology, Loyola University Maryland, Baltimore, MD, United States

**Keywords:** insomnia, history, Sleep Psychology, Behavioral Sleep Medicine, treatment outcomes studies

## Abstract

In the 1980s, Patricia Lacks (February 22, 1941–December 3, 2016) began a systematic program of research at Washington University in St. Louis to identify the causes of insomnia and effective treatments. Her treatment outcomes research culminated in the publication of the first detailed cognitive-behavioral treatment manual for insomnia, *Behavioral Treatment for Persistent Insomnia*. This paper focuses on the history of Dr. Lacks and her contributions to the field of insomnia research and practice.

## 1. Background

Patricia (Pat) Lacks was born Patricia J. Everett in Oregon on February 22, 1941; months before Pearl Harbor and the entrance of the United States into World War II. Due to her father's role in the Air Force, her family moved frequently during her childhood. She lived in over a dozen places throughout the U.S. and abroad in Japan and Brazil. She was a dedicated student. Drawn to science early in life, she confronted negative attitudes toward women early and often in her academic pursuit of it. She viewed psychology as a serious science and obtained her B.S. in Psychology and Ph.D. in Clinical Psychology at Washington University in St. Louis. In 1966, when she obtained her Ph.D., women represented only 1/5 of those earning doctoral degrees in psychology (Scarborough, 1992). Her dissertation investigated differences in stimulus generalization among psychiatric patients (Brilliant, 1966, 1968). After completing her doctorate, Dr. Lacks worked for Washington University for 20 years, while raising her 2 children. She also maintained a part-time private practice treating insomnia, depression, and obesity. Dr. Lacks worked for many years as an adjunct faculty member before entering the tenure track. In collaboration with Amy Bertelson Ph.D., she developed a program of research around insomnia treatment outcome studies. Her interest in insomnia grew, in part, out of her own struggles with sleep, including sleep difficulties associated with parenting young children. Her insomnia work was part of a Zeitgeist of sleep research that occurred in the St. Louis area in the 1980s, including sleep research being conducted by colleagues at Washington University, the newly established Deaconess Sleep Disorders and Research Center in St. Louis, and St. Louis University. It was an exciting and productive time for her, her colleagues, and her students. However, she expressed difficulty in obtaining research funding for her psychological work on insomnia (Lacks, 1987). Although by the 1980s women were equally represented among those receiving advanced degrees in psychology, women psychologists at Dr. Lacks' career stage remained underrepresented in leadership and status (Scarborough, 1992). There were few women in the Psychology Department at Washington University and Dr. Martha Storandt was the only tenured woman on the faculty at that time. Dr. Lacks and others felt that there was an atmosphere of unfriendliness toward professional women. There was also a sense that her family commitments, including leaving work each day to provide childcare, were viewed unfavorably. After 7 years of dedicated insomnia research, she experienced a lack of recognition for the merits of her work from the Department of Psychology and was denied tenure, much to the dismay of many of her peers and students. She remained in the department as an adjunct faculty member for several years until her children graduated from high school, at which time she retired from Washington University. She moved to California with her husband, where she worked as a clinician for The Sleep Disorders Center of Santa Barbara. She also taught classes at UC Santa Barbara and Antioch University. She spent much of her adult life with her third husband, Paul Gawronik. After a 10-year battle with Alzheimer's disease, she died at the age of 75.

## 2. Contributions to the field of sleep research and practice

### 2.1. Emphasis on the psychological aspects of insomnia

Dr. Lacks was a brilliant psychologist, accomplished writer, beloved teacher, skilled clinician, and caring mentor. She made notable contributions to the field of clinical psychology, including her book *Bender Gestalt Screening for Brain Dysfunction* (Lacks, 1984, 1999). At the time, it was the first major manual on Bender Gestalt assessment in over 30 years. She also published articles in the scientific literature in the areas of psychological assessment and treatment (Brilliant and Gynther, 1963; Gynther and Brilliant, 1967; Lacks, 1982; Lacks and Storandt, 1982; Levine et al., 1983).

Dr. Lacks' career shifted to insomnia in 1980, during a time when conceptual definitions of insomnia were nascent and diverse. A year earlier the Association of Sleep Disorders Centers (ASDC) published a nosology of sleep and arousal disorders that classified insomnia-related disorders (insomnias) under the “Disorders of Initiating and Maintaining Sleep (DIMS)” classification, one of the 4 provisional categories (Association of Sleep Disorders Centers the Association for the Psychophysiological Study of Sleep, 1979). The 1980 edition of the *Diagnostic and Statistical Manual for Mental Illnesses* included the ASDC nomenclature in the appendix (American Psychiatric Association, 1980)— a reflection perhaps of the growing recognition that mental or psychological aspects play a role in sleep disorders. Initially, she viewed insomnia as a subjective symptom (Lacks, 1987). For Dr. Lacks, the unifying factor of the DIMS was that they resulted in a final common pathway of sleeplessness, which for her included disturbed sleep (i.e., difficulty going to sleep, difficulty remaining asleep, and difficulty going back to sleep when awakened in the night) and diminished sleep (i.e., short sleep duration). Although the ASDC nomenclature parceled out 9 DIMS subcategories, she believed that psychological treatment was most appropriate for the subclassification of subjective insomnia and psychophysiological insomnia. She noted that many instances of insomnia have physiological origins requiring medical evaluation and treatment. With the publication of the *International Classification of Sleep Disorders* in 1990 (American Sleep Disorders Association Diagnostic Classification Steering Committee, 1990), her perspective evolved with the field in viewing insomnia as a diagnosable disorder, albeit a heterogeneous one. Shedding tangential correlates of insomnia (e.g., short sleep duration), her final publication on insomnia adopted an empirical definition of insomnia as involving “sleep onset latency or wake time after sleep onset >30 min/night, a minimum of 3 nights per week), chronic (at least 6 months), and produces a complaint of daytime impairment (fatigue, performance, and mood decrements)” (Lacks and Morin, 1992). Despite the evolution in the nosology of insomnia over the years, her research and clinical approach latched onto the core aspects of insomnia that remain relevant today.

While much of the sleep research and clinical treatments of insomnia prior to 1980 viewed insomnia as a physiological disorder and focused largely on pharmacological interventions, Dr. Lacks emphasized the psychological aspects of insomnia. She spearheaded an effort spearheaded an effort to manualize psychological treatments for insomnia and experimentally test their efficacy and effectiveness using randomized control trials. The results of her treatment outcome studies for insomnia appeared in 1983 (Lacks et al., 1983a,b; Puder et al., 1983). That same year, *Sleep Disorders: Basic and Clinical Research* was published (Chase and Weitzman, 1983), in which the section on insomnia treatment focused entirely on pharmacological options and failed to mention non-pharmacological interventions at all. In stark contrast, Dr. Lacks recommended withdrawal from sleep medication as a prerequisiteprerequisite for beginning psychological treatment for insomnia. She tirelessly highlighted the risks, common problems, and limited utility of sleep medications for the treatment of persistent insomnia; even going as far as to suggest that helping clients gradually withdrawal from sleep aids can resolve the original sleep problem. To help understand the psychological factors in treatment seeking individuals with insomnia, one of her studies used the MMPI to investigate how personality differed in those with sleep onset insomnia from controls (Levin et al., 1984). Results broadly replicated previous studies that compared good sleepers to poor sleepers or individuals with chronic insomnia symptoms, showing that insomnia is associated with neurotic personality features including greater anxiety, more concern with bodily sensations, depressed mood, feelings of inadequacy, inferiority, and lack self-confidence (Monroe, 1967; Chauvin et al., 2015).

### 2.2. Publication of the first CBTI manual

Dr. Lacks seminal contribution was the publication of the first detailed clinical guidebook for insomnia assessment and treatment, *Behavioral Treatment for Persistent Insomnia (Lacks*, *1987*). Designed to train the non-specialist, this publication represented the culmination of her knowledge, experience, and wisdom acquired during her 6 years of treatment outcome research on insomnia. Her clinical guidebook reads like a scholarly article, thorough in its summary of the extant literature and evidence-based in its recommendations. Although the title of the book might appear behaviorist in orientation, a closer read reveals a cognitive-behavioral approach that was nuanced, comprehensive, and incorporating biological, environmental, behavioral, and cognitive factors. Her work built on over 20 years of prior research that had been conducted on non-pharmacological treatments for insomnia. To produce the manual, she pulled from and credited the work of other psychologists in the field including Drs. Richard Bootzin, Peter Hauri, Thomas Borkovec, Kenneth Lichstein, Charles Morin, James Walsh, Collin Espie, Bernie (Bernie) Webb, and many other scholars in sleep science to effectively summarize for the current and next generation of clinicians the core information required to understand, assess, and treat insomnia competently. To create a comprehensive program, she connected the reader with the client's experience, created assessment measures, incorporated multiple components into a cohesive treatment program, and outlined session scripts with descriptions and helpful pointers for how to effectively administer the treatment, much of which that has stood the test of time.

### 2.3. Insomnia characterization

In her treatment manual, she provided a characterization of a typical individual with chronic insomnia, while preserving nuance for each individual case. She recognized the importance of individual differences in demographics (age, sex, employment, socioeconomic status), physiology, mental health, personality styles, experiences, attitudes, preferences, substance use, and culture context when conceptualizing the cause of insomnia and treating it. She highlighted the importance of the cognitive, experiential, and phenomenological aspects of insomnia to its etiology and treatment. For Dr. Lacks that there were at least 5 factors that contributed to some degree in most individuals with insomnia: somatic arousal, emotional arousal, performance anxiety, self-efficacy, and cognitive arousal (Cook and Lacks, 1984). Her book appeared in the same year as Spielman and colleagues' articles that describe sleep restriction and the 3P model of insomnia (Spielman et al., 1987a,b). Dr. Lacks too, described how behavioral and cognitive changes in response to acute sleep difficulties are key to the vicious cycle of insomnia. She described a typical sequence of insomnia precipitation and maintenance as (1) a temporary sleep problem; (2) resulting in worry about being able to sleep and consequences of insufficient sleep; (3) changes in thinking patterns exacerbating sleep difficulties; (4) unpleasant feelings and cognitive arousal become associated with the bed and bedroom; (5) behavioral changes in response to sleep loss including napping, curtailing daytime activities, going to bed early, and sleeping in, result in erratic sleep patterns; (6) self-efficacy erodes, daytime impairments increase including feelings of inadequacy and depression; (7) often in desperation recourse to sleeping aids fails; and (8) finally after over a decade the “person's life becomes one large exacerbation cycle and downward spiral” before reaching an insomnia therapist (Lacks, 1987, pp. 56–57). Her description of the lived experience of individuals with insomnia remains palpable, including the social stigma of insomnia, the neglect they experience from the medical community, the loneliness endured during the night and feeling like they are the only ones unable to sleep, the dismissal of insomnia problems by friends and family, and the dislike of being labeled “insomniacs.”

### 2.4. Development of insomnia assessments

Dr. Lacks emphasized a scientist-practitioner approach to treating insomnia involving detailed assessment of the clients' personal, sleep, psychological, and medical history using a semi-structured clinical interview, daily sleep diaries, and questionnaires. The semi-structured clinical interview and sleep diary she published within the manual were used and modified by subsequent insomnia researchers (Fichten et al., 2001, 2005). Although several versions of the sleep diary had been widely used in sleep research and clinical practice for decades (Monroe, 1967), to our knowledge, Dr. Lacks published the first manuscript that specifically focused on the sleep diary—outlining its nebulous history/origins and its clinical use in insomnia (Lacks, 1988). Recognizing the importance of accurate prospective data in guiding treatment, she provided her participants with stamped and addressed envelopes to mail their sleep diaries back to the clinic each day, similar to procedures used in other labs at the time (Lick and Heffler, 1977). Weekly assessments of adherence to treatment recommendations were emphasized to guide the intervention, improve adherence, and reinforce and solidify treatment gains. She also recommended psychological measures (e.g., the Beck Depression Inventory and State-Trait Anxiety Inventory) and insomnia-specific questionnaires be used before and after treatment to assess the sleep problem and measure intervention effectiveness (Lacks, 1987). She believed that assessment of pre-sleep cognitive/somatic arousal, self-efficacy, and sleep hygiene were key to effective treatment. To assess the latter 2 constructs, she developed two novel questionnaires.

First, recognizing the importance of cognitive factors in insomnia etiology and treatment outcomes, she introduced the concept of self-efficacy to sleep research (Cook and Lacks, 1984). Self-efficacy, as described by psychologist Albert Bandura, Ph.D. (Bandura, 1977), refers to an individual's belief about their ability to do something. Dr. Lacks argued that low self-efficacy and high-performance anxiety around sleep were often primary elements in the maintenance of insomnia and key to its treatment. She describes the paradox of insomnia, in which individuals with insomnia have a strong desire for sleep, they often have superior knowledge about sleep, and yet seem to prioritize sleep less in their behavior (Lacks and Rotert, 1986). A major aim of her approach was to increase self-efficacy and turn the participant into a “personal scientist” with greater self-awareness and capability of using skills and behavioral experiments to improve their sleep. Her statement to clients that “Very little that is worth having in life comes without effort. Again, we reiterate that long standing habits cannot be changed without consistent and persistent effort” (p. 120) encapsulates her emphasis on self-control in her treatment approach. Dr. Lacks believed that the therapist's ability to increase self-efficacy was key to the treatment, making it superior to self-help books available at the time. She developed the Self-Efficacy Scale, which included items on individuals' beliefs about their ability to carry out treatment recommendations and reach sleep-related goals (Lacks, 1987, p. 79). Based on her clinical experience and the research with this scale, she concluded that a change in self-efficacy around sleep was one of the most consistent and profound impacts of her treatment for insomnia. She stated, “The most frequent expressed outcome by clients in our program has been the transformation from feeling one is a helpless victim to a sense of being in charge and control again (Lacks, 1987, p. 88). The Self-Efficacy Scale was later validated in older adults (Fichten et al., 2001) and remains in use to the present (Ghose et al., 2022). Furthermore, in the scholarship of one of her student's, sleep-health efficacy has been shown to play a role in both parenting and infant sleep and in early adolescents' sleep health (Wolfson et al., 1992, 2015).

Second, influenced by the work of Peter Hauri, Ph.D., sleep hygiene was an important component of her approach. Sleep hygiene advice was extant at the time, but she recognized the importance of providing evidence-based recommendations and tailoring them to specific cases. Her team published the first sleep hygiene questionnaire for use in individuals with insomnia: The Sleep Hygiene Awareness and Practice Scale (SHAPS) (Lacks, 1987). This three-part questionnaire assessed (1) general knowledge of sleep hygiene, (2) knowledge of what substances contain caffeine, and (3) sleep hygiene practices. Although requiring revision to address psychometric limitations, SHAPS has been useful in gaining a better understanding of insomnia, guiding insomnia interventions, and informing subsequent measures of sleep hygiene (Brown et al., 2002, 2006; Spielman et al., 2003; Berger et al., 2009; Yang et al., 2010; Otte et al., 2016). Her work using this questionnaire, along with many since, support the current clinical position that addressing sleep hygiene factors may be best viewed as necessary, rarely sufficient for the resolution of insomnia (Lacks and Rotert, 1986).

### 2.5. Promotion of multi-component treatments for insomnia

Dr. Lacks treatment manual offered a complete multi-component treatment program for insomnia. Stimulus control (Bootzin, 1972), an operant conditioning method wherein sleep is thought to reward pre-sleep behaviors, was the core feature of Lacks' treatment approach, one that she considered most important. However, she seemed to view stimulus control more as a therapeutic procedure than a technical behaviorist construct. Far more than that found in the description of Bootzin (1972) and Bootzin and Nicassio (1978), Dr. Lacks rationale for stimulus control provided to clients has a ring of classical conditioning:

*Much of what we do is influenced by the time and the place we are in. The stimulus, or characteristic of a stimulus gets paired with the behavior that occurs in that situation the characteristics of the situation then become a signal or cue for that behavior…For people who have insomnia, the bed and bedroom may have become a signal for other activities…After the disruption or stress is over, poor sleep, frustration, and any activities that the poor sleeper may have performed while waiting for sleep (e.g., reading, watching television) remain associated with the bed and the bedroom. Subsequently, the bed remains a cue for these nonsleeping behaviors and for being awake…With stimulus control you will learn to reassociate the bed and the bedroom with rapid sleep onset. The bed and bedroom will be weakened as cues for other activities. You will learn to maximize the cues that are associated with feeling sleepy and falling asleep, and to decrease the cues that are associated with staying awake”* (Lacks, 1987, pp. 95–96).

Of note, is the absence of any mention of sleep itself as a reward for pre-sleep behavior. Indeed, one of her other publications questioned whether reestablishing the bed as a discriminative stimulus for sleep was the active ingredient in stimulus control (Davies et al., 1986). She also noted that some aspects of standard stimulus control procedures used in clinical trials do not necessarily fit within an operant paradigm. Reflecting her nuanced understanding of insomnia and its treatment, she separated several of the traditional stimulus control procedure and listed them as sleep hygiene recommendations in her manual including don't go to bed until sleepy, get up at the same time each morning, and do not take naps.

The other major components included in her manual were (1) the therapist, (2) group process, (3) sleep education, (4) self-monitoring, (5) self-control, (6) reduction of performance anxiety, and (7) sleep hygiene. In addition, she incorporated several other elements into her treatment approach, including establishing a pre-bed routine, encouraging exercising, scheduled worry time away from the sleep environment, relaxation, and medication withdrawal. For older adults she recommended they wake up at the same time each morning and “keep regular schedules of eating, meeting friends, volunteering their time, taking walks, and any other potential activities” to “regularize [the] sleep rhythm [and] promote an active, less sedentary, stimulating life that will serve to enhance sleep” (Lacks, 1987, pp. 128). Although sleep restriction was not included as a distinct element, she too recognized the importance of restricting time in bed to the individual's sleep need, at a regular time of day (Lacks, 1987). Inspired by Albert Ellis' work, her approach was also strikingly cognitive involving promoting the treatment with persuasion, normalizing insomnia, reducing mental arousal, reframing, counterdemand instructions including emphasizing that things are likely to get worse before they get better and that improvements should not be expected until the fourth week, encouraging problem-solving, contrasting valuation of long- vs. short-term sleep goals, discouraging clockwatching, discriminating factors associated with good and poor nights of sleep using the sleep diary, eliciting commitments from clients, fostering self-efficacy, and utilizing cognitive control strategies. Dr. Lacks proposed that other stand-alone treatments designed to address cognitive arousal may be beneficial to clients as well (Davies et al., 1986), and recommended that cognitive refocusing (Zwart and Lisman, 1979) and progressive muscle relaxation (Bernstein and Borkovec, 1973) be administered in “successive sieves,” following stimulus control in waves, abbreviated by a consolidation period between each treatment (Lacks, 1987, pp. 122–125). Although her manual did not include cognitive restructuring, it emphasized both behavioral and cognitive components in her conceptualization and treatment of insomnia. Thus, *Behavioral Treatment for Persistent Insomnia* represents the first published cognitive-behavioral therapy for insomnia (CBTI) manual.

### 2.6. Group treatment for insomnia

To our knowledge, Dr. Lacks developed the first intervention for insomnia that incorporated group process as a treatment component. She believed that insomnia treatment was best conducted in the group setting, with group size ranging from five to seven members (Lacks, 1991). She argued that the group setting helped address client's feelings of being alone in their insomnia and fostering peer pressure and support within the group. Her group approach to insomnia treatment was directive and exacting (e.g., intolerant of non-adherence). It was not, however, rule-governed; rather, she focused on applying the principles of the treatment known to work and adapting the sleep recommendations to each sleeper's motivations and circumstances. For example, she provides insightful recommendations for adapting the treatment to older adults for optimal effects, including providing age-relevant psychoeducation, adapting material presentation to accommodate age-related impairments in vision and hearing, spending more time on important treatment components, and simplifying the treatment and logs to essential elements. She also recognized that the approach outlined in her manual could be readily applied to individual therapy and provided valuable insights in ways her approach could be adapted to different therapeutic styles.

### 2.7. Treatment outcome studies and insomnia research

Along with other research teams conducting treatment outcome research before and during the 1980s, Dr. Lacks and her colleagues revealed that non-pharmacological treatments for insomnia are efficacious (Lacks and Powlishta, 1989). Her team investigated the relative impact of several types of insomnia interventions including stimulus control, progressive relaxation, paradoxical intention, sleep hygiene, and countercontrol (Lacks et al., 1983a,b; Davies et al., 1986; Schoicket et al., 1988). These non-drug studies were among the largest and best controlled at the time, allowing them to helped establish stimulus control as an evidence-based treatment for insomnia, including for those with sleep maintenance problems (Espie, 1991). She also helped debunk myths about treatment of insomnia in older adults. In contrast to the view that older adults with insomnia should be treated pharmacologically or in their own group setting, she demonstrated that independent and educated older adults do in fact respond to stimulus control (Puder et al., 1983) and that they can be treated together with young adults in insomnia group treatment (Davies et al., 1986). Dr. Lacks was down-to-earth, eschewing a dogmatic theoretical orientation, and focused instead on empirical evidence and practical wisdom. A true critical thinker, she had incredible integrity, looking beyond “statistical significance” to honestly assess the clinical significance of her work. She concluded that only about 50% of participants in her 7 treatment studies experienced reliable changes (Lacks, 1987). Showing greater concern for the improvement of society than that of her book sales, she acknowledged the limitations of her research and treatment approach. She encouraged the development of new evidence-based and more comprehensive multicomponent treatments to improve insomnia outcomes (Lacks and Morin, 1992).

## 3. Legacy

Dr. Lacks developed the first detailed treatment manual for insomnia, providing a structured and practical approach to clinically manage insomnia (Lacks, 1987). Her work contributed to the foundation on which modern CBTI and evidence-base practice for insomnia was built in subsequent decades (Perlis and Lichstein, 2003). During her active research years, she was well-recognized and well-respected in the discipline for her treatment studies research (K. Lichstein, personal communication). Her invitation to write the insomnia review for the *Journal of Consulting and Clinical Psychology* was an honor and reflects the respect she garnered. Her research, particularly her early studies supporting stimulus control as an efficacious treatment for insomnia, are also well recognized in the literature and historical accounts of Behavioral Sleep Medicine (BSM) (Stepanksi and Perlis, 2003; Stepanski, 2003; Morin et al., 2006). Her manual and most of her insomnia research articles were cited and well-represented in subsequent foundational guidebooks for insomnia assessment and treatment (Espie, 1991; Morin, 1993). She is also currently remembered as being ahead of her time in her emphasis on sleep self-efficacy in insomnia treatment. However, some aspects of her historical contributions to the field of insomnia have been overlooked, including recognition for weaving a cognitive orientation into stimulus control treatment, thereby publishing the first CBTI manual, scripting a classical conditioning description of stimulus control widely used by therapists today, and introducing group therapy as a component of insomnia treatment. In the History of the Development of Sleep Medicine in the United States published in in the *Journal of Clinical Sleep Medicine*, Dr. Bootzin's stimulus control and the treatment studies conducted by Dr. Lacks and others showing its efficaciousness were not mentioned in the history of insomnia treatment research (Shepard Jr et al., 2005). To our knowledge, Dr. Lacks has not been recognized *via* awards for her contributions and this is the first publication acknowledging her as a pioneering woman in the field of insomnia research and sleep medicine.

The venues through which her work was published (i.e., books, psychology journals, and psychological conferences, as opposed to sleep journals and sleep meetings) and the circumstances of her employment at Washington University (i.e., limited funding for large-scale treatment studies and shortened research career due to tenure denial) likely limited her visibility in the field. There is also evidence “that women's scientific contributions are systematically less likely to be recognized” across disciplines (Ross et al., 2022), and this cannot be ruled out as a contributing factor. Based on interviews with those close to her at the time, her decision to promote her work outside of the sleep community was influenced, at least in part, by her perception that the field of sleep medicine was male-oriented. The annual Association for the Psychophysiological Study of Sleep (APSS) meetings, which evolved into the SLEEP conferences in 1986, were well attended during the peak of Dr. Lacks' career. However, she did not attend because she did not feel that women were well recognized. While the American Psychological Association was attentive to the issues and significant role of women in the profession as early as 1973, evidenced by the founding of the Committee on Women in Psychology (CWP) (Scarborough, 1992), the first recognized organization in the sleep community, Women in Sleep and Rhythms Research (WiSRR) was not acknowledged until the mid-1990s, after Dr. Lacks' time. Her identity as a psychologist may have also played a factor in her conference selection and publication venues. The rebranding of the Sleep Research Society in the 1980s removed “psychophysiological” from its name; this was perhaps symbolic of psychology's place in the evolving field of sleep medicine. Like many psychologists doing insomnia research at that time, she promoted her work at psychology conferences including at the Association for Advancement of Behavioral Therapies, which limited her interactions with other sleep scientists. Nevertheless, her psychological perspective had an impact on contemporary and subsequent insomnia researchers. For example, Dr. Walsh one of her former colleagues described how her work and arguments persuaded him to begin to consider the psychological aspects of insomnia and its treatment (J Walsh, personal communication).

During her research career, Dr. Lacks mentored, taught, and supervised many undergraduate and graduate students, including Amy R. Wolfson, Ph.D. (author), who would go on to make many important contributions to the field of sleep and circadian rhythms, particularly in children and adolescents. As a mentor, Dr. Lacks was supportive, encouraging, enthusiastic, efficient, and demanding of rigor (Behrendt, 1978; Cook, 1985; White Cook, 1985; Schoicket, 1986; Wolfson, 1987). Most of her insomnia-related publications included student co-authors, whom she described as being more like colleagues than research assistants (Lacks, 1987). She encouraged her students to develop a relentless curiosity, be willing to make mistakes, and pursue one's passion—advice she modeled in her own life (Wolfson, 2015). Moreover, she was deeply devoted to mentoring her women graduate students in their scholarship, clinical work, and career advice. For example, she was known for reminding them that “women can have it all: career, friends, and family, but never more than two of the three successfully at the same time” (RD Sulser, personal communication). Another former student noted that she “was one of our best professors in that she prepared us to be clinicians and teachers; She was also an excellent role model for me as a woman” (A Hauger, personal communication).

Many in the current generation may be unaware of the extent to which Dr. Lacks' clinical approach to insomnia has influenced its current treatment. Nevertheless, for those who know her work, she is an inspiration, particularly to women in the field of insomnia research and clinical practice. As a woman, a psychologist, and an insomnia researcher, she was a pioneer in sleep research, particularly in the areas that would become Behavioral Sleep Medicine and Sleep Psychology. Although perhaps not identifying as a “sleep researcher” during her time, she is a welcome guest at the banquet table of sleep research in the annals of history.

## Data availability statement

The original contributions presented in the study are included in the article, further inquiries can be directed to the corresponding author.

## Author contributions

DK wrote the first draft of the manuscript, and conducted the background research including a literature review and interviews. AW conducted a background literature review and interviews, provided personal insights, and helped write and edit the manuscript. All authors contributed to the article and approved the submitted version.
